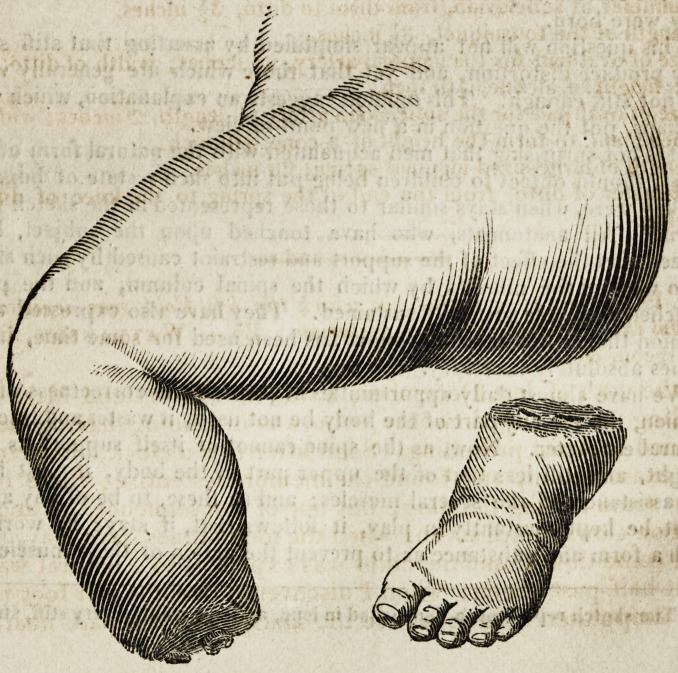# Case of a Fœtus, in Which the Left Foot Was Separated from the Leg during Utero-Gestation

**Published:** 1825-07

**Authors:** 

**Affiliations:** Surgeon, &c.


ART. IX.—*Case of a Fœtus, in which the left Foot was separated from the Leg during Utero-gestation.* By — WATKINSON, Esq. Surgeon, &c.

ON the 29th of last December, I was called to attend Mrs. —, residing at —, aged twenty, who was in labour, having been married in April preceding. Soon after five o'clock, I made my first examination, and found the membranes entire; there had been some trifling flooding, but not of consequence. Labour went on slowly until seven, when the membranes gave way, the head came down, and the child was expelled, in a natural way, about half-past seven; when I discovered that the left foot had been amputated a little above the ankle, and the part nearly, but not quite, healed, in consequence, perhaps, of the bones protruding. The child was alive, and gasped for twenty minutes, when it expired. The mother said she had only gone seven months, which perfectly agreed with the appearance of the infant. On examination after the birth, I discovered the foot in the vagina, which I brought away: it also was nearly healed, but here likewise the bones protruded. There did not appear to have been any extravasation of blood from the limb, as far as I was able to judge. This foot (the left) was much smaller than the other, which was rather turned inward: it showed no marks of putrefaction, and, from comparing the two, I am led to suppose that it must have been two months separated from the body. There was not the slightest discoloration of the foot, which was in a state of perfect preservation.

The mother says, she was not frightened, nor did any unpleasant circumstance occur in the family during her pregnancy, to give her the least uneasiness. The husband is a journeyman, and by his industry they live comfortably, without her having to use more exercise than is actually necessary in a well-regulated family.

*King-street, Soho; March* 1825.

---

***** We have examined the preparation, and subjoin a rough sketch of the parts. —EDITORS.**

## Figures and Tables

**Figure f1:**